# Immunity for nothing and the eggs for free: Apparent lack of both physiological trade-offs and terminal reproductive investment in female crickets (*Gryllus texensis)*

**DOI:** 10.1371/journal.pone.0209957

**Published:** 2019-05-15

**Authors:** Atsushi Miyashita, Ting Yat Marco Lee, Laura E. McMillan, Russell Easy, Shelley A. Adamo

**Affiliations:** 1 Department of Psychology and Neuroscience, Dalhousie University, Halifax, Nova Scotia, Canada; 2 Department of Biology, Acadia University, Wolfville, Nova Scotia, Canada; University of Vienna, AUSTRIA

## Abstract

Should females alter their reproductive strategy when attacked by pathogens? Two hypotheses provide opposite predictions. Terminal reproductive investment theory predicts that reproduction should increase when the risk of death increases. However, physiological trade-offs between reproduction and immune function might be expected to produce a decrease in reproduction during a robust immune response. There is evidence for both hypotheses. We examine whether age determines the effect of an immune challenge on reproductive strategy in long-winged females of the Texas field cricket, *Gryllus texensis*, when fed an ecologically valid (i.e. limited) diet. The limited diet reduced reproductive output. However, even under resource-limited conditions, immune challenge had no effect on the reproductive output of young or middle-aged females. Both reproductive output and immune function (lysozyme-like activity and phenoloxidase (PO) activity) increased with age, which is contrary to both hypotheses. We hypothesize that PO activity is pleiotropic and represents an investment in both reproduction and immune function. Three proPO genes (identified in a published RNA-seq dataset (transcriptome)) were expressed either in the fat body or the ovaries (supporting the hypothesis that PO is bifunctional). The possible bifunctionality of PO suggests that it may not be an appropriate immune measure for studies on immune/reproductive trade-offs. This study also suggests that the threshold for terminal reproductive investment may not decrease prior to senescence in some species.

## Introduction

Resources are finite for all organisms. How resources are allocated across growth, somatic maintenance, and reproduction is an important determinant of fitness. Reproduction and immunity are two metabolically expensive traits [1]. Activation of an immune response can lead to physiological trade-offs, resulting in declines in reproduction (e.g. insects [1–3]; vertebrates [4–8]; plants [9]). Exposure to pathogens likely signals an environment with a high pathogen prevalence [10] leading to enhanced immune function (e.g. immune priming [11–13]). This shift in resource investment in favor of immune function should lead to a long-term reduction in reproductive output. However, under some conditions, an immune challenge leads to increased reproductive output, which is usually interpreted as a type of fecundity compensation [14]. This response is called terminal reproductive investment [15,16], in which the immune-challenged organism shifts investment away from somatic maintenance to fuel a final bout of reproduction prior to death. Whether an individual should increase or decrease reproduction when immune-challenged depends on a range of poorly understood factors (e.g. age [14]).

Age is expected to reduce the fitness benefit gained from increasing immune function and decreasing reproduction (i.e. a physiological trade-off) when infected [14]. Residual reproductive value declines with age [17], and, therefore, the fitness payoff for reducing current reproduction to preserve future reproduction should decline over time. Furthermore, if declining condition due to age (i.e. during senescence) reduces the chance of recovery (e.g. [18]), then it may be adaptive for older organisms to prioritize reproduction during an immune challenge. Therefore, age should increase the likelihood that terminal reproductive investment will be activated by an immune challenge [14]. Insects make good model systems for these types of questions, in part because their reproductive output is easy to quantify, and their immune systems are simpler than those of vertebrates [19]. Moreover, insects like the cricket *Gryllus texensis* are large enough to measure multiple immune components simultaneously (e.g. key insect immune components such as phenoloxidase (PO) activity [20]).

Supporting the hypothesis that age affects the reproductive response to infections, interactions among age, immune challenge and reproductive investment have been observed in several male insect models such as *Gryllodes sigillatus* [21], *Drosophila nigrospiracula* [22], and *Allonemobius socius* [23]. However, evidence in female insects is relatively scarce, even though reproductive output in females is often easier to quantify than it is in males. Immune challenge has a variable effect on female crickets (Table 1), possibly because the effect is sensitive to female age. In *G*. *texensis*, females retain high immunocompetence throughout their adult stage, while it declines in males [18]. This result suggests that females may have a reproductive resource allocation strategy that differs from that of males (also see [24] for sex-specific effects of macronutrient intake on trade-offs between reproduction and immunity in the cricket *G*. *sigillatus*). We examine the effect of age on the long-term effects of pathogen exposure on both reproductive output and immune function. We hypothesized that a challenge early in life should signal increased pathogen prevalence, leading to a long-term shift towards reduced egg laying and increased immune investment. We predicted that older females, however, should not upregulate immune function after challenge, but instead increase egg laying.

**Table 1:** Effect of immune challenge on reproduction in female crickets.

Species	Dosage	Age (post adult) at the start of treatment	Duration of immune challenge	Effect on reproduction	Reference
*Acheta domesticus*	100 μg/cricket of *Serratia marcescens* LPS	2 weeks	Acute	+ (positive)	[25]
*Acheta domesticus*	5 x 10^4^ live cells of *S*. *marcescens*	2 or 5 weeks	Acute	+	[25]
*Acheta domesticus*	1 or 2 nylon pieces/cricket (implantation)	18 days	Chronic (3 weeks)	- (negative)	[26]
*Gryllus texensis*	8.75 x 10^3^ live cells of *S*. *marcescens*	11 to 19 days	Acute	+	[17]
*Gryllus texensis*	1 x 10^5^ live cells of *S*. *marcescens*	11 to 19 days	Acute	0 (no effect)	[17]
*Gryllus texensis*	1.2 x 10^5^ live cells of *S*. *marcescens*	2 weeks	Acute	+	[17]
*Gryllus texensis*	LD_01_ of *S*. *marcescens* live cells	2 weeks	Acute	0	[27]
*Gryllus texensis*	LD_01_ of *Bacillus cereus* live cells	2 weeks	Acute	-	[27]
*Gryllus texensis*	20 μg/cricket of *S*. *marcescens* LPS	13 to 19 days	Chronic (every three days for 12 days)	0	[28]
*Gryllus texensis*	100 μg/cricket of *S*. *marcescens* LPS	13 to 19 days	Chronic (every three days for 12 days)	0	[28]
*Gryllus texensis*	1 x 10^4^ heat-killed *S*. *marcescens*	1 day	Chronic (every three days for 17 days)	-	[29]
*Hemideina crassidens*	100 μg *S*. *marcescens* LPS	Uncontrolled (field collection)	Chronic (every four days for 17 days)	-	[30]
*Hemideina crassidens*	500 μg *S*. *marcescens* LPS	Uncontrolled (field collection)	Chronic (every four days for 17 days)	-	[30]
*Gryllodes sigillatus*	Nylon implantation (encapsulation response)	14 day	Chronic	0	[24]

Unfortunately assessing immune function is problematic [31]. Immune function is made up of multiple components, which can sometimes be negatively correlated with each other [32]. Immune systems can also reconfigure their molecular network pathways, and therefore a reduction in a single immune component may be mistaken for a reduction in investment, as opposed to a reconfiguration [33]. Finally, the primacy of different immune pathways can shift depending on the physiological context [33–36]. Therefore, to monitor immunological investments in crickets, it is important to measure multiple aspects of immune function on each animal. We measured phenoloxidase activity (PO), glutathione concentration (GSH, which helps buffer the self-damage caused by PO [37]) and lysozyme-like activity in the hemolymph. PO and lysozyme-like activity respond differently to immune challenges; lysozyme-like activity is inducible in response to pathogen challenge, while PO is a constitutive component of immune defense in insects [38].

Although PO activity is commonly used as a proxy for immune function in ecoimmunological studies, PO is also involved in egg production in insects. PO contributes to the tanning of the egg chorion [39,40] and/or the eggs' antimicrobial defense [41,42]. This complicates the interpretation of PO levels in female insects. In *G*. *texensis*, PO activity in eggs has also been reported [29]. There appears to be multiple sources of PO in insects, and this may depend on the species. Although hemocytes have been viewed as a major source of PO [20,43,44], in some insects, the fat body and the ovaries also express POs (e.g. mosquitoes, see Fig 5 of [45]). Little is known in insects about how these POs are trafficked between organs, thus it remains unclear whether the hemolymph PO level reflects either immune investment or reproductive investment, or both. Therefore, we also assessed PO gene expressions in both fat body and ovaries.

In this study, we will also monitor flight muscle maintenance. In a closely related cricket (*Gryllus firmus*), adult crickets histolyze their flight muscles over time [46], but the pace of histolysis is slower in the long-wing (flight capable) morph than in the short-wing (flightless) morph. In *G*. *firmus*, all individuals in the short-wing morph histolyze their flight muscles by day 5–7, whereas long-wing morphs histolyze their wing muscles at a variable age, often past day 12 [46]. Within the long-wing morph of *G*. *firmus*, females with histolyzed muscles show an increased reproductive output compared to those with functional muscles, suggesting a competitive resource allocation between flight and reproduction. In our study, we use the long-wing morph of *G*. *texensis*. We monitor whether wing muscle histolysis is more frequent in immune challenged crickets.

## Materials and methods

### Animals

Female *G*. *texensis* crickets were originally obtained from San Antonio, Texas, USA, and have been maintained in the laboratory for approximately 8 generations. The colony was maintained at 26°C on a 12/12 hour light/dark cycle, supplied with food (cat food pellets, ‘No Name Balanced Nutrition for Cats’ by Loblaws Inc. (Toronto, Canada)) and water *ad libitum*. Long-winged adult females were weighed and isolated from the colony within 48 hour after the imaginal molt (the day that we call 'day 1' in this study). Each of the females was isolated in a plastic container and supplied with a shelter and a water bottle (diameter = 2 cm, height = 6 cm). Upon isolation (day 1), food was intermittently provided in the individual containers for 3 hours every 3 days, unless otherwise stated. During those 3 hours, crickets could feed *ad libitum*. This intermittent feeding condition has been shown to produce females with the same fat content as females collected in the field [47]. On days 7 and 8 (female adult age), each female was provided with three different males. Each male was placed in the female’s container for about 8 hours. Males were switched between containers so as to ensure that each female was exposed to three different males. At the end of the 36-day experimental window, crickets were dissected. Females that had translucent spermatheca (i.e. empty of sperm) were excluded from the study (8 out of 240 crickets were excluded from the analysis for this reason). All experiments were approved by the Animal Care Committee of Dalhousie University (# I-11-025) and are in accordance with the Canadian Council on Animal Care.

### Treatments

To examine the effect of age on the reproductive response to infection in the cricket *G*. *texensis*, we chose two age classes: young (11 days old as an adult) and middle-aged (21 days old). At 11 days of age (i.e. young crickets), females have mated and some have begun to produce eggs, but their reproductive activity (oviposition rate) is less than maximal compared with older crickets [17]. By 21 days of age, females are fully mature, with high oviposition rates in most female individuals [17]. Either age class is within the typical age for females found in the field [48]. We did not include crickets older than the plausible lifespan in the field (i.e. 4 to 5 weeks in the field [48]). Behavioural traits observed in unrealistically old individuals have little impact on fitness in the field.

**Fig 1:**
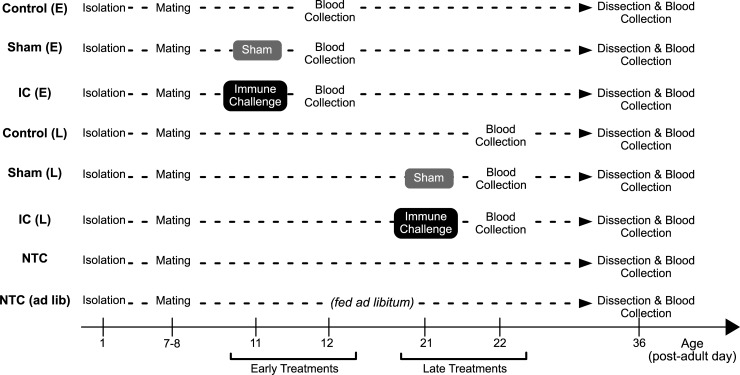
Experimental schedules. Each cricket used in this study (except for ones for the ovarian gene expression experiment) went through one of the eight experimental timelines. The horizontal axis shows the experimental timeline (day). IC: Immune Challenge, Sham: Sham injection, NTC: No-Treatment Control.

Female crickets were first sorted by body weight on day 1, and assigned to one of the following eight treatments (see below). To equalize body size across groups, the heaviest cricket was assigned to group 1, the next heaviest to group 2, etc.; in the next cohort, the heaviest was placed in group 2, next heaviest to group 3, and so on. Days were counted from the day of isolation (i.e. post-adult day; see timeline in Fig 1). Injections (and sham injections) were performed by inserting a needle (of a 10 μL Hamilton syringe) into the body cavity under the pronotum plate. Assessment of reproductive output and our hemolymph collection protocol are described after the description of the different treatment groups.

Below is a detailed description for each of our 8 treatments: Control crickets (Early Treatment Control (E) or Late Treatment Control (L)), Sham-treated crickets (Early Treatment Sham (E) or Late Treatment Sham (L)), and Immune Challenged (IC) crickets (Early Treatment IC (E) or Late Treatment (L) IC). Treatment differences across the groups are summarized in Table 2.

*Early Treatment Controls (Control (E))*. Crickets were handled on day 11, and hemolymph samples were collected on days 12 and 36.
*Late Treatment Controls (Control (L))*. Crickets were handled on day 21, and hemolymph samples were collected on days 22 and 36.
*Immune Challenge–Early Treatment (IC (E))*. On day 11, crickets were injected with 2 μL of a mixture of heat-killed pathogen cells *(Serratia marcescens*, *Bacillus cereus* and *Beauveria bassiana*.) Bacteria were obtained from Carolina Biological (Microkwik cultures, Burlington, NC, USA) and the *Beauveria bassiana* was BotaniGard 22WP (Laverlam, Butte, MT, USA). The dose of each pathogen was adjusted to approximately 1/10 of the LD_50_ dose prior to heat inactivation (*S*. *marcescens*, 2 x10^4^ cells; *B*. *cereus* 2 x 10^3^ cells; *B*. *bassiana*, approximately 1 x 10^4^ cells). The heat-killed bacteria treatment is known to activate immune responses in this species [38]. We added the fungi, also known to activate immune responses in related insects (e.g. Mormon crickets [49]), to increase the breadth of the challenge. Hemolymph samples were collected on days 12 and 36.
*Immune Challenge-Late Treatment (IC (L))*. Crickets given an immune challenge on days 21 with 2 μL of the same heat-killed pathogen mixture described above. Hemolymph samples were collected on days 22 and 36.
*Sham-Early Treatment (Sham (E))*. On day 11, crickets were poked with a 10 μL Hamilton syringe, but were not injected. Hemolymph samples were collected on days 12 and 36.
*Sham-Late Treatment (Sham (L))*. On day 21, crickets were poked with a 10 μL Hamilton syringe, but not injected. Hemolymph samples were collected on days 22 and 36.
*No Treatment Control (NTC)*. Crickets were not handled or sampled throughout during the 36 day trial. Hemolymph samples were collected on day 36.
*No Treatment Control (ad lib feeding) (NTC (ad lib))*. Crickets were fed ad lib for 36 days. Hemolymph samples were collected on day 36.

**Table 2:** List of treatments given to control, Sham, and IC crickets.

	Wound caused by blood collection on day 12 or 22	Poking by a needle on day 11 or 21	Pathogen injection on day 11 or 21
NTC*			
Control	+		
Sham	+	+	
IC**	+	+	+

* No Treatment Control

**Immune Challenged

### Reproductive output

The reproductive output of each female was monitored by counting the number of eggs laid, the number of eggs still remaining in the lateral oviducts on day 36, and egg quality (e.g. hatch rate).

To count the number of laid eggs, females were given cotton balls (Fisher Scientific, #07–866 ‘Non-Sterile Absorbent Cotton Balls’) placed in a water bottle. The water bottle served as an egg-laying substrate and as a water supply. We replaced the water bottle with a new one (with new cotton balls) twice a week after mating. Total number of laid eggs between day 12 and day 36 and day 22 and 36 was calculated (Eggs were typically collected on days 12, 15, 19, 22, 25, 29, 33, and 36). Eggs were collected immediately before the blood collections (i.e. day 12 or 22).

Eggs in the lateral oviducts were counted as follows. Crickets still alive on day 36 were dissected after being weighed. For each cricket, we counted eggs in the lateral oviducts. During the dissections, we observed the state of the flight muscles (functional/histolysed as described by Zera [50]).

Egg quality was assessed as follows. On each egg collection day, five eggs were subsampled from each cotton ball and placed separately in centrifuge tubes (1.5mL) with a small piece of cotton and 500 μL of water. In cases where the number of eggs laid in the cotton ball was less than five, all eggs were sampled. These eggs were then kept at 26°C and monitored for 35 days. Hatch date, hatchling survival (daily), and hatchling body mass at 35 days after the hatch day were monitored for the sampled eggs. Eggs were censored and assumed not viable if they had not hatched within 35 days. The reproductive value (RV), a proxy for fitness, was calculated as a product of the number of eggs and the hatch ratio. For example, if a female laid 50 eggs and 3 out of the 5 subsampled eggs hatched, then the reproductive value would be 30.

### Hemolymph collection

We collected hemolymph samples by poking the membrane under the pronotum plate with an ice-cold pipette tip (to retard coagulation), and the hemolymph was collected as it exited the wound. We collected 8 μL of hemolymph which was mixed with 55 μL of ice-cold MilliQ water in a 1.5 mL centrifuge tube. (We used water instead of PBS, because it disrupts cells, allowing us to assess PO in both plasma and hemocytes). Samples were then split into three fractions (20 μL for the PO and Bradford assays, 23 μL for the GSH assay, and the rest (20μL) for the Lysozyme assay). Immediately after the sample collection, we spun the hemolymph sample for the GSH assay (23 μL) at 18,800 g for 10 min at 4°C, and 20 μL of the supernatant was immediately mixed with 20 μL of 100 mg/mL meta-phosphoric acid. After 5-minute incubation at room temperature, we spun the samples at 2,900 g for 3 min at room temperature. Thirty-five (35) μL of the deproteinated supernatant was collected in a new 1.5 mL centrifuge tube. All samples for PO, Bradford, GSH, or Lysozyme assays were stored at -8°C until use.

### Hemolymph assays

Total PO activity and total protein concentration were measured as described previously [38]. GSH concentration was measured as described previously [51]. Detailed information for the assays is described in S1 Methods. Below is a brief description of each method.

PO Activity and Total Protein Concentration were determined as follows. Samples were thawed and mixed with 34 μL of reverse-osmosis filtered water (RO water), then spun at 10,000 rpm for 5 minutes at room temperature. Twenty-eight μL of the supernatant was mixed with 28 μL of 2 mg/mL chymotrypsin (Sigma, #C7762-100MG), and incubated for 20 minutes at room temperature. The incubated sample was then spun at 10,000 rpm for 5 minutes at room temperature, and 15 μL of the supernatant was mixed with 180 μL of saturated L-3,4-dihydroxyphenylanaline (L-DOPA) solution (118mg powder (Sigma, #D9628-25G) suspended in 30 mL RO water). Fifteen (15) μL of RO water was run as a blank. A standard curve was determined using tyrosinase (Sigma, #T3824-25KU) standards at 450, 45, 22.5, 4.5, 2.25, 0.90, and 0.18 μg/mL. Wells were measured at OD_490_ every 30 seconds for 30 minutes, and Vmax was estimated. Mean values from triplicate measurements were used for the analysis. The remainder of the hemolymph sample (26μL) was used to measure its protein concentration with a Bradford assay kit (Sigma, #B6916-500ML), following the manufacturer’s instructions.

GSH was measured as follows. We used a commercial kit (Cayman Chemical, #703302) for GSH measurements. After thawing deproteinated hemolymph samples, we processed the samples according to the manufacturer’s instructions. We used the protocol for measuring total (i.e. both reduced and oxidized forms of) GSH.

Lysozyme-like activity was measured as follows. Hemolymph samples were collected as described above. Samples were thawed and spun at 12,000 g for 3 min at 4°C. Five μL of the supernatant was mixed with 45 μL of *Micrococcus luteus* cell (Sigma #M3770) suspension (10 mg/20 mL Phosphate-buffered Saline (PBS), pH = 7) in a 96-well (flat bottom) plate. The mixture was incubated at 30°C, and we measured OD_450_ every 30 seconds for 50 minutes. Lysozyme derived from chicken egg white was used to produce a standard curve (Sigma-Aldrich, #62971-10G-F). PBS was used as a reference blank. The mean value from triplicate technical replicates was used for each sample.

### Gene identification

To identify the gene transcripts in the cricket, we first constructed a transcriptome database based on a raw sequence of RNA reads available online (National Center for Biotechnology Information (NCBI, https://www.ncbi.nlm.nih.gov/). The accession number of this project is PRJNA429132 (submitted by Natural History Museum, Berlin, Germany). We then set up a searching pipeline (written in Python programming language, the code is available at the author's GitHub repository at https://github.com/atmiyashita/CricketGeneFinder2018/). The code: (1) fetches cDNA sequences in arthropods from NCBI Nucleotide database that are associated with the target protein name (i.e. 'vitellogenin', 'phenoloxidase' etc.), (2) runs BLAST locally using the fetched sequence as a query and the transcriptome (of *G*. *texensis*) as a database, (3) outputs the result in xml format, and (4) returns a summary. The hit sequences were then assessed by blastx at https://blast.ncbi.nlm.nih.gov/Blast.cgi?PROGRAM=blastx&PAGE_TYPE=BlastSearch&LINK_LOC=blasthome to confirm its homology at amino acid sequence level (i.e. primary structure). For vitellogenin, we further performed a physiological validation in this study (S3 Fig), because the sequence similarity was relatively low (compared to proPO, see S1 Fig).

### Gene expression analysis

For gene expression analysis in the ovaries, we dissected a group of females that were independent of the rest of the study and collected both the fat body and the ovaries. These crickets were also given the intermittent diet during adulthood. The primers used in this study are listed in S1 Table. We followed the MIQE guideline [52,53] for the qPCR experiments. Detailed information for RNA extraction, cDNA synthesis, and quantification is described in S1 Methods. The fat body (the speckled white tissues found in the abdominal cavity) was collected carefully to minimize collecting other tissues such as the trachea. The ovaries were collected carefully so as not to contaminate the sample with fat body. We washed ovaries once with PBS to further avoid potential contamination of the sample with hemocytes. The tissues were stored in 300 μL of RNAlater (Thermo Fisher Scientific, #AM7020) in 1.5mL centrifuge tubes and frozen at -8°C until further use.

### Data analysis

Statistical tests were done using R version 3.5.2 [54]. In this study, we isolated 240 female adult crickets assigned across 8 treatment groups. Eight out of the 240 crickets did not mate (i.e. 232 crickets contained spermatheca filled with sperm when dissected). These 8 were excluded from the analysis. Sixty-six (66) crickets were also excluded from the analysis because of missing data (e.g. due to death, S2 Fig). Thus, we acquired a complete dataset on 166 female crickets that mated and survived for 36 days. As a measurement of condition, the Scaled Mass Index (SMI) [55] was calculated for each cricket. Cricket size was determined by calculating the average length of the hind leg femurs. Femur length is a good estimate of total body size [56]. The calculation formula for SMI is as follows [57]:

$$Scaled\;Mass\;Index\;(SMI)≔M_{i}{\left(\frac{L_{0}}{L_{i}}\right)}^{b_{SMA}}$$

where M_i_ and L_i_ are the body mass and the linear body measurement (i.e. femur length) of i-th individual; b_SMA_ is the scaling exponent estimated by the standard major axis regression of the body mass and the linear size; L_0_ is the arithmetic mean on the femur lengths for the study population.

To test the effect of feeding condition on reproductive output, we performed Mann-Whitney U tests comparing NTC and NTC (ad lib) groups. We performed three statistical tests on (1) the number of total produced eggs, (2) that of laid eggs, and (3) that of eggs found in the lateral oviducts. Significance level in these statistical tests were corrected by Benjamini-Hochberg procedure to control for multiple tests on the same data set.

To test the effect of immune challenge on reproduction, we used generalized linear models, assuming negative binomial errors for the response variable (i.e. egg counts). We chose this model because the distribution of the observed egg counts was non-normal (right skewed) and over-dispersed (the variance was approximately 100 times larger than the mean). Non-normality was tested by Shapiro-Wilk test using ‘shapiro.test’ function in R. We compared Akaike Information Criterion (AIC) of the model containing treatment effects and an AIC of its null model (containing no independent variable). Tested models are listed in S2 Table. Because all full models showed higher AICs than the corresponding null models (S2 Table), we did not perform further post-hoc tests on parameter estimates. We used ‘glm.nb’ function in ‘MASS’ package in R. Details for the modelling are described in S1 Methods.

To test the effect of immune challenge of hemolymph parameters, we used generalized linear mixed models, in which we treated the cohort number (i.e. experimental replicate) as a random effect. We chose this model because the observed immune measures showed right-skewed distributions with large variances. Non-normality of the distributions were tested by Shapiro-Wilk test. Tested models are listed in S3 Table. If the AIC of the model containing treatment effect is lower than that of its null model, then we performed post-hoc z-test to test whether the coefficient of the fixed effect (treatment effect) is non-zero. We performed eight post-hoc tests in this analysis (see S3 Table), and the significance levels were corrected by Benjamini-Hochberg procedure to control the false discovery rate at 0.05. We used ‘glmer’ function in R. Survival analyses (shown in S2 Fig) were performed using ‘survival’ package on R. Details for the modelling are described in S1 Methods.

To examine the effects of age alone on hemolymph immune measures, we used a subset of data: (1) the hemolymph data on day 12 from Control (E) group, (2) the hemolymph data on day 22 from Control (L) group, and (3) the hemolymph data from day 36 from the NTC group. All of these crickets experienced blood collections for the first time in their life on either date (see Fig 1). In other words, we did not use the hemolymph data from crickets that had experienced a blood collection or immune challenge (Sham or IC) by the blood sampling dates to exclude effects other than age (e.g. immune priming). For example, we did not use day 36 data from Control (E/L) because they had already experienced a blood collection on day 12 or day 22. We used generalized linear mixed models, in which we treated the cohort number as a random effect. We assumed gamma distributions for the response variable with a long link function. Tested models are listed in S4 Table. If the AIC of the model containing treatment effects is lower than that of its null model, then we performed a post-hoc analysis to test whether the coefficient is non-zero. We performed four post-hoc tests in this analysis (S4 Table), and the significance level was corrected using the Benjamini-Hochberg procedure to control for multiple tests. Details for the modelling are described in S1 Methods.

To examine the difference of gene expression between tissues, we performed Mann-Whitney U tests. The crickets used in this analysis (11 females) are independent from other analyses in this study. We performed four statistical tests on different target genes, and the significance levels were corrected by Benjamini-Hochberg procedure to control for multiple tests.

## Results

### Effect of food availability on overall reproductive output

**Fig 2:**
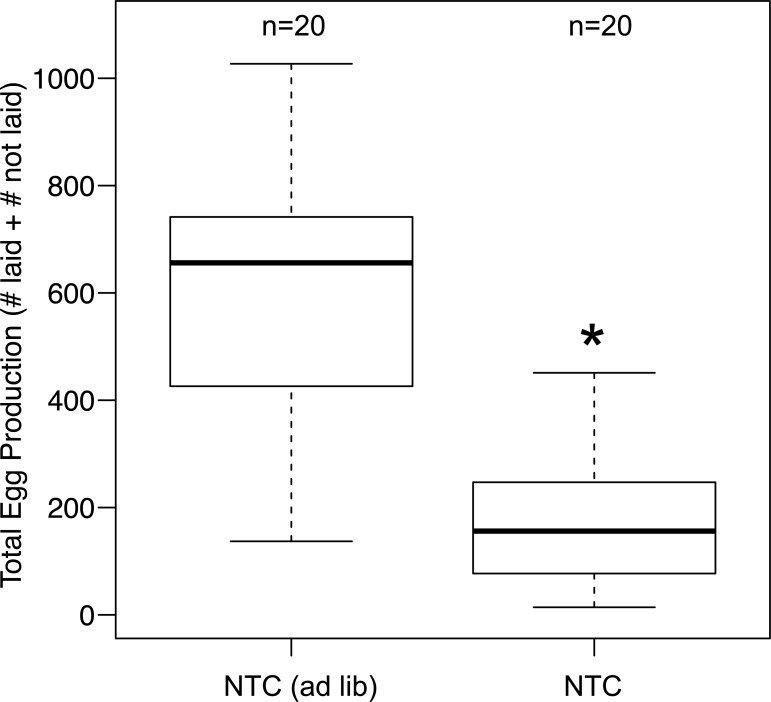
Effect of feeding condition on reproductive output. NTC crickets (food limited) produced fewer eggs (laid + not laid) by day 36 compared with NTC (*ad lib*). The bars represent the 25^th^ and 75^th^ percentile, the central line in bold represents the median and the error bars denote the maximum and minimum values for each group. Statistical information is described in the Results section.

We tested whether food limitation (i.e. intermittent feeding) reduced resource availability for reproduction in female crickets. Controls that were food-limited (NTC) produced fewer eggs by day 36 than controls fed *ad libitum* (i.e. (NTC (*ad lib*)) (Fig 2, Mann-Whitney U test: p = 1.0x10^-5^), suggesting that the intermittent feeding condition limited the resources available for reproduction. The food limited females also laid fewer eggs (Mann-Whitney U test: p = 0.0011) and contained fewer eggs in the lateral oviducts (Mann-Whitney U test: p = 7.1x10^-4^) (S4 Fig).

### No effect of immune challenge on reproductive output

**Fig 3:**
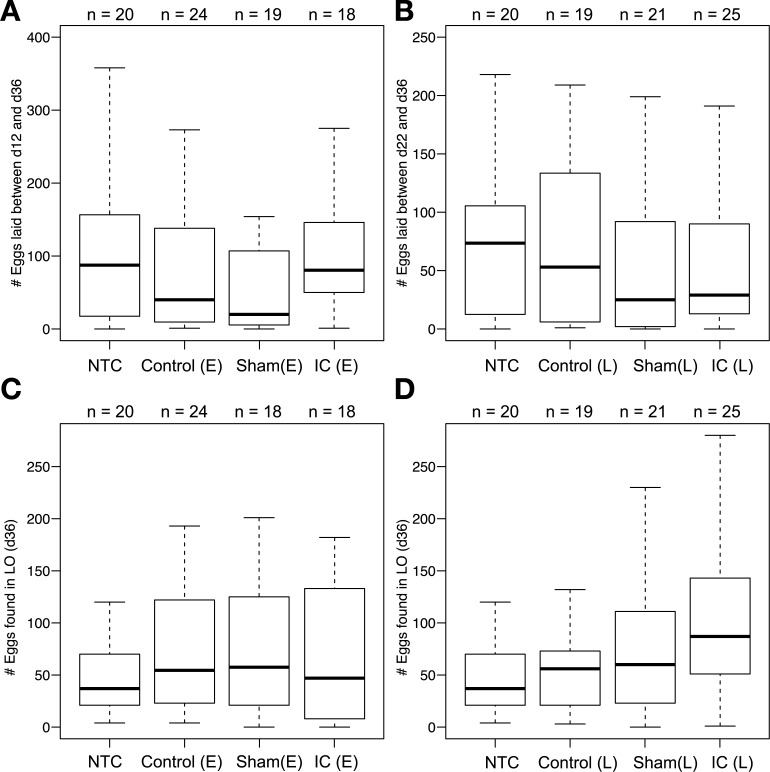
No effect of immune challenge on reproductive output. The number of eggs laid between day 12 and 36 (A), day 22 and 36 (B), and the number of eggs found in the lateral oviducts (C and D) are shown across treatment groups. The bars represents the 25^th^ and 75^th^ percentile, the central line in bold represents the median and the error bars denote the maximum and minimum values for each group. Statistical information is described in the Results section. No significant difference was found among groups.

Immune challenge on either day 12 or 22 did not increase the number of eggs laid (Fig 3A and 3B, and S2 Table). Also, there was no effect of immune challenge on the number of eggs in the lateral oviducts (Fig 3C and 3D, and S2 Table). If an effect of immune challenge on the number of eggs laid existed, it would require more than 1700 crickets/group to find, suggesting a small, and probably biologically insignificant effect at best (S5 Fig).

Also, there was no difference in the reproductive value (RV; see Methods for details) at any time point across groups (S6 Fig). Eighty-seven percent (130 out of 150 observations) of the females had already histolysed their flight muscles by day 36, and this occurrence was equal across groups (Fisher’s exact test: p = 0.75). Nevertheless, the females that had histolysed the flight muscles by day 36 showed higher reproductive outputs that those that had not (S7 Fig), suggesting a link between dispersion capability and reproductive output.

### Effect of immune challenge on immune parameters and survival

In young females, immune challenge (IC (E)) decreased PO activity (S3 Table). Sham treatment (Sham (E)) increased the protein level (S3 Table). In middle-aged females, IC (L) showed a trend toward a significant positive effect on the lysozyme-like activity. None of the treatments showed a long-lasting effect on these measures (i.e. significant on day 36) (S3 Table, S8 Fig).

The overall survival rate on day 12 was 100% (210/210), on day 22 it was 99% (207/210), and on day 36 it was 79% (166/210) as shown in S2 Fig. There was no difference in survival across the seven groups (S2B Fig; χ^2^ = 9.8, df = 6, p = 0.13), confirming that the immune challenge was sublethal.

### Age-dependent changes in immune measures

**Fig 4:**
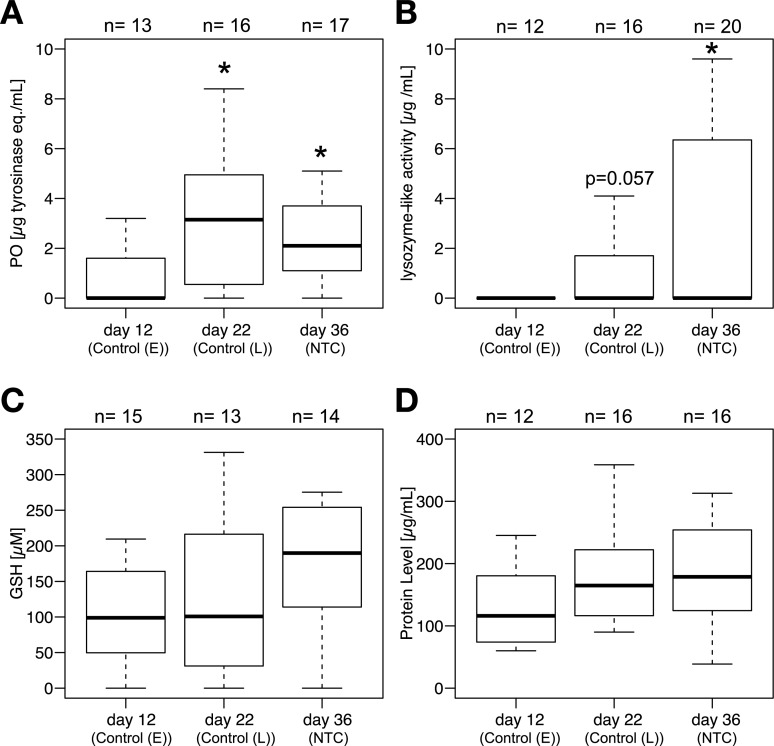
Age-dependent increase of PO and Lysozyme level. Age-dependent change in hemolymph immune measures. (A) PO level (μg (tyrosinase equivalent)/mL), (B)lysozyme-like activity (μg/mL), (C) GSH concentration (μM), and (D) protein concentration (μg/mL) are shown in the chart. Data for day 12 are from Control (E), data for day 22 are from Control (L), and data for day 36 are from NTC. activity and the lysozyme-like activity were increasing with age (A-B), while GSH and the protein concentrations did not show a significant trend (C-D). Statistical information is described in the Results section. The bars represents the 25^th^ and 75^th^ percentile, the central line in bold represents the median and the error bars denote the maximum and minimum values for each group. Sample sizes are indicated above each plot.

In females that did not experience immune challenge or sham injection (i.e. Control (E), Control (L) and NTC), PO and lysozyme-like activity increased with age (Fig 4A and 4B; S4 Table), while GSH and protein concentration did not show significant difference across ages (Fig 4C and 4D, S4 Table). Also, reproductive output between day 22 and 36 (4.7 eggs/day) was higher than that between day 12 and 22 (2.2 eggs/day) (Wilcoxon signed rank test: n = 146, V = 2042, p = 8.1x10^-9^; also see S6 Fig).

### Expression of proPOs in the ovaries

**Fig 5:**
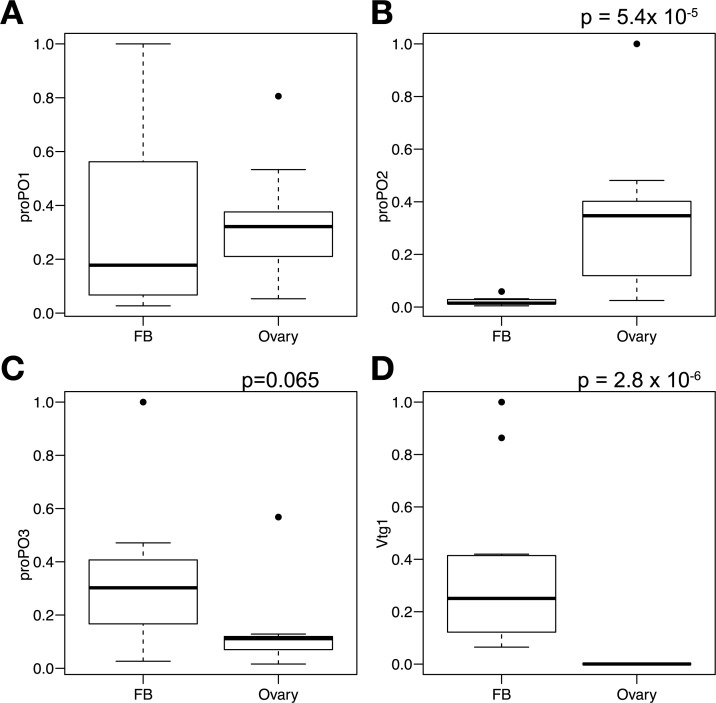
Ovaries produce phenoloxidase (PO) in the cricket. Gene expression levels of *proPOs* (A-C) and *vitellogenin* (D) were measured in the fat body and the ovaries. The values were normalized by two reference genes, and the relative expression levels (arbitrary units, where the maximum expression level is set at 1.0 for each transcript) are shown in the figure. The bars represents the 25^th^ and 75^th^ percentile, the central line in bold represents the median and the error bars denote the maximum and minimum values for each group. Outliers plotted as dots. Statistical information is described in the Results section. The sample size (biological replicates) is N = 11. FB: Fat Body.

Three *proPO* transcripts were expressed in fat body and ovaries. *proPO1* was consistently expressed in the fat body and the ovaries at comparable levels (Fig 5A, Mann-Whitney U test: p = 0.75). *proPO2* was expressed specifically in the ovaries (Fig 5B, Mann-Whitney U test: p = 5.4x10^-5^), while *proPO3* was expressed in both tissues (Fig 5C, Mann-Whitney U test: p = 0.065). Vitellogenin was expressed only in the fat body (Fig 5D, Mann-Whitney U test: p = 2.8x10^-6^).

## Discussion

We report three major findings: (1) Immune challenge had no long-term effects on reproduction in either young or middle-aged female crickets (Fig 3). (2) Egg production (S6 Fig), and the activity of two major immune components (Fig 4), increased with age, suggesting a positive relationship between these two traits. (3) Genes for different POs were expressed in both fat body and ovaries (Fig 5), suggesting that PO in the hemolymph may be involved in both reproduction and immune function in crickets. Females in this study were short on resources; the females on the food limited diet laid fewer eggs than those fed ad lib (Fig 2). Therefore, these results suggest that resource-strapped females funnel resources into both egg production and immune function at the end of life, which is inconsistent with both a physiological trade-off [2] and the terminal investment hypothesis [14].

We do not believe that these results are due to technical issues. For example, one possible alternative explanation is that the bleeding caused by blood collection suppressed the potential positive effect of immune challenge on reproduction. However, given that NTC (No treatment control) and Control (Blood collection only) groups showed comparable reproductive output, the reproductive cost of handling and bleeding appears to be too low to have had an effect on reproductive output. Another possibility is that the immune challenge used was insufficient to induce terminal reproductive investment. However, the same bacterial challenge has been shown to induce a robust immune response in this species in this lab [38,58], as well as sickness behaviour [59], and short-term terminal reproductive investment [17]. It could be argued that the animals challenged at 22 days were too young to show terminal reproductive investment. This species can survive for more than 8 weeks in the laboratory [17]. However, *G*. *texensis* females show signs of senescence after only 4 weeks [17], which is probably close to their maximum lifespan in the field [48]. A 4 week ecological lifespan is consistent with our study that showed an increase in mortality only at the last time point (i.e. 36 days, S2 Fig). Only 6.7% of females (n = 104) collected in the field were 22 days or older in this species [17]. This field result suggests that by 22 days, the crickets are entering their final decadal age cohort. Therefore, these animals were appropriately old from an ecological context. Our final collection day was day 36, the beginning of senescence. At this age, terminal reproductive investment theory [60] suggests that maintaining high levels of investment in immunity should reduce fitness, given that females are short on resources and likely to die prior to being felled by an infection.

Interestingly, the increase in PO with age is consistent with an earlier study that found an age-dependent increase in PO activity in female, but not in male, *G*. *texensis* [18]. An increase in PO activity with age has also been found in females of other species (e.g. *Tenebrio molitor* [61]). In old female *T*. *molitor* this increase in PO leads to Malpighian tubule damage, suggesting that the increased PO activity was a sign of immune dysregulation [61]. However, in this study, females maintained a constant PO:GSH ratio across ages (S4 Table). GSH helps buffer the self-damaging toxicity of PO [37]. Therefore, even at age 36, we suspect that high PO is not a sign of immune dysregulation.

The increase in PO activity with age in some female insects may not represent an increase in immune investment, but may represent an increase in reproductive investment. PO is needed for the tanning and defense of insect eggs [39–42]. In some insects, it appears to be synthesized by sources outside of the ovary and transported to the ovaries through the blood (e.g. mosquito [62]). Therefore, increases in PO hemolymph levels may represent an increase in reproductive effort, which concomitantly also increases the amount of PO available for immunity. We have three lines of evidence for this in our study: (1) PO activity rises in middle-aged (day-22) crickets, which is prior to senescence (S2 Fig), (2) Middle-aged females have a high reproductive output, consistent with an increased need for PO to maintain increasing egg production (S5 Fig), and (3) the age-dependent increase in PO is observed only in female *G*. *texensis* [18], suggesting that the rise of PO is involved with female-specific life-history traits such as egg production. Furthermore, mating enhances disease resistance in *G*. *texensis* females [63]. Mating also triggers egg production in crickets [64]. Therefore, mating leads to an increase in both egg production and immune function in this species. These paradoxical results could be explained by the dual-functionality of immune molecules such as PO.

We detected proPO gene expression in the fat body, which could supply PO to both hemolymph and ovary. However, we also found that the ovary expressed two subtypes of proPO genes, and, therefore it is uncertain to what extent the ovary and fat body contribute to egg PO (and to the hemolymph PO levels that we measured). We have not done an in-depth molecular analysis of the proPOs expressed in the ovaries, but the differential expression of proPO subtypes between the fat body (primarily an immune organ, but also involved in production of yolk protein (i.e. vitellogenin [65]), and the ovary demonstrates the complexity and pleiotropic nature of PO. Identifying the circulating PO subtype(s) in the hemolymph and in the eggs would help answer this question. It is these types of mechanistic details that are needed to understand trade-offs (e.g. [14,66]). This complexity also suggests that PO is not an ideal proxy for immune investment in immune/reproductive trade-off studies in some female insects. Many molecules in animals are multifunctional [67], leading to pleiotropic effects that complicate the study of trade-offs.

An increase in lysozyme-like activity with age could be a function of the increased likelihood of pathogen exposure with age. Baseline lysozyme-like activity increases after pathogen exposure in other cricket species (e.g. [68,69]). In our study, most females had very low levels of lysozyme-like activity, even on day 36 (Fig 4). However, by day 36, a significant number of crickets had high levels, possibly because they had fought off an infection at some point in their lives. The longer a cricket lives, the more likely it is that it will need to respond to a pathogen. It is unclear how long lysozyme-like enzymes exist in the hemolymph. In humans, lysozyme has a short half-life in plasma (less than an hour [70]). However, in insects, lysozyme-like activity can be increased for days after an infection (e.g. crickets [68,69]), but whether this is from increased synthesis, or maintenance of standing levels is unknown. There is evidence for upregulated lysozyme gene expression during infection in insects (e.g. *Manduca sexta*[71]; *Trichoplusia ni* [10]). Therefore, the increase in lysozyme-like activity with age may simply reflect historical exposure to pathogens. In support of this, a similar increase in lysozyme-like activity with age has been found in male crickets (*Acheta domesticus* [69]), suggesting that the increase is not tied to female reproduction.

Although we measured a number of immune components, we did not assess the entire immune system of the cricket. For example, we did not assess cellular immune components. Others have found a trade-off between lysozyme-like activity and cellular immune responses [72–74]. Therefore, it is also possible that the increase in lysozyme-like activity is part of a reconfiguration of the immune system, with a change in emphasis between different immune compartments with age. Such a reconfiguration occurs during food limitation in the caterpillar *M*. *sexta* [33].

Most ecoimmunological studies in crickets are performed on either long-winged or short-winged morphs, because the morphs are known to have different reproductive strategies [75]. However, a sub group of long-winged crickets in *G*. *firmus* can histolyze their wing muscles at different times over their adult life [46,75], and, as this paper suggests, in *G*. *texensis* as well. Histolyzing wing muscles releases additional resources for reproduction in other cricket species [46,76]. Once the muscles are histolyzed, they are no longer capable of flight [77]. The additional resources provided by the wing muscles may reduce trade-offs between immunity and reproduction. This presents an interesting complexity regarding allocation of resources in this sub group of female crickets. These females can alter their investment in somatic maintenance, reproduction and/or dispersal during their adult life. The investment in dispersal, although not-all-or-none, is not incremental in the same way that immune and reproductive traits can be. It appears that once the commitment is made to histolyze the flight muscles, they cannot be rebuilt (see [46]), even if dispersal might be an effective strategy at a later date.

Previous studies have found evidence of terminal reproductive investment in crickets [17]. However, those immune challenges were close to a lethal dose. The effect of a sub-lethal dose of bacteria on reproduction was only observed in *G*. *texensis* when moist sand was used for egg-laying substrate, and was not observed when moist-cotton was used, as in this study [17]. *G*. *texensis* females prefer to oviposit in moist sand over moist cotton [17], which may have affected the terminal investment thresholds. This point needs to be validated in future studies.

This study also suggests that the threshold for terminal reproductive investment may not decrease prior to senescence. We did not test for terminal reproductive investment during senescence (i.e. post 28 days), but the decline in body condition that occurs during senescence could be a powerful cue for terminal reproductive investment. Prior to senescence, the risk of death for female crickets is likely to be the same each day, assuming the same pathogen and predator prevalence. Therefore, there may be little selection to alter their investment strategy until their condition wanes (i.e. senescence). The lack of effect of age on whether infection triggers the terminal reproductive investment threshold reflects the generally weak effect of age on the reproductive response to infection in other female insects [14,25,78,79] and females from other phyla[14].

## Supporting information

S1 Methods Supplementary information for the methods of this study. (DOCX)

S1 Table List of Primers for qPCR. (DOCX)

S2 Table Summary of generalized linear models for the effect of immune challenges on reproductive output. (DOCX)

S3 Table Summary of generalized linear mixed models for immune measures. (DOCX)

S4 Table Summary of generalized linear mixed models for immune measures over age. (DOCX)

S1 Fig Sequence information. Target genes (transcripts) were identified as described in Materials and Methods. Illustrated here is the sequence query (translated to amino acid sequence) and typical hit results in NCBI’s BLAST search. (A) vitellogenins. (B) proPOs.
(DOCX)

S2 Fig Survival. Survival of crickets. 210 crickets across eight groups (see Materials and Methods) were monitored daily (A). There was no significant difference in survival across the eight groups (B).
(DOCX)

S3 Fig Physiological validation of vitellogenin transcripts. Physiological validation of the three vitellogenin transcripts (vitellogenin 1, 2, and 3). Normalized expression level in the fat body in females on day 1 (F01), day 12 (F12), day 22 (F22), day 36 (F36) and males on day 12 (M12). Each plot represents an individual cricket (biological replicate). The values in the y-axes represents relative expression levels (arbitrary units), where the expression level for the reference genes is set to be 1.0.
(DOCX)

S4 Fig Effect of food limitation on reproductive output. The number of eggs laid by day 36 (A) and the number of eggs found in the lateral oviducts on day 36 (B) are shown in the chart. Food limited NTC crickets produced fewer eggs compared with ad lib fed NTC crickets The bars represents the 25th and 75th percentile, the central line in bold represents the median and the error bars denote the maximum and minimum values for each group. Statistical information is described in the Results section. (A) Mann-Whitney U test: p = 0.0011. (B) Mann-Whitney U test: p = 7.1x10-4. Sample size is n = 20 for each group.
(DOCX)

S5 Fig Power analysis of the statistical test for the effect of immune challenge on reproduction. We found no significant effect of immune challenge on the reproductive output of female crickets. The parameter estimates in the generalized linear mixed model were: Dispersion parameter for Negative Binomial 0.5187 β for Intercept (β_0) 4.2966 β for IC(L) effect (β_(IC(L))) -0.1302 Using these parameter estimates for gamma distribution, we performed a power analysis by simulation. We iterated 200 random samplings from two gamma distributions (the μ of distribution 1 is exp(4.2966) and that of distribution 2 is exp(4.2966–0.1302)) with different sample sizes (10 to 3000). We calculated the power by dividing the number of positive instance by 200. In other words, if 80 out of 200 iterations found significant difference, then the power is 0.4. The y-axis of the figure represents the calculated power, and the x-axis represents the sample size. As shown in the figure, more than >1700 crickets were required to obtain a power of >0.8. Attached below is the code in R for this computation.
(DOCX)

S6 Fig Time course of reproductive value (RV) (by treatment group). Time course of RV. The reproductive value (RV), a proxy for fitness, was calculated as a product of the number of eggs and the hatch ratio. It provides a rough estimation of the number of offspring for each female. For example, if a female laid 50 eggs and 3 out of the 5 subsampled eggs hatched, then the reproductive value would be 30. Each black line represents an individual cricket. The red lines (with dots) represents median values at each time point. The RVs are log10-transformed. The total sample size for the crickets shown above is 136, because 10 of 146 crickets lacked hatchling data, and, thus, were not possible to obtain RVs. Below is a list of treatments.
(DOCX)

S7 Fig Dispersion capability may be traded off with reproduction. Most crickets had white (histolysed) flight muscle on day 36 (114 of 131 observations; 5 females lacked muscle observation data). Nevertheless, given that the retention of pink (functional) muscle is associated with dispersion capability of crickets (which is one of the important life-history traits in this species), we performed an additional (i.e. post hoc) analysis to examine the association between the dispersion capability and the reproductive outputs. We excluded the NTC (ad lib) group from this analysis because we did not note wing muscle colour in this group. The crickets that still retained pink (functional) flight muscles on day 36 (n = 17) produced and laid fewer eggs than the crickets with histolysed (white) flight muscle (n = 114). Mann-Whitney U test: W = 303, p-value = 5.2x10-06. The bars represents the 25th and 75th percentile, the central line in bold represents the median and the error bars denote the maximum and minimum values for each group. 15 of 146 crickets were excluded from this analysis, because they lacked muscle observation data.
(DOCX)

S8 Fig Humoral responses to immune challenge. Hemolymph biochemistry parameters are shown in the chart. The columns represent measurements on days 12, 22 or 36. The rows represent PO, GSH, Lysozyme-like activity, and the total protein levels. The treatment effects were examined by generalized linear mixed models (using ‘glmer’ function in 'lme4' package in R), considering cohort (i.e. experimental replicate) as a random factor in the model. The y-axes represent PO level (μg (tyrosinase equivalent)/mL), the GSH level (μM), the lysozyme-like activity (μg (egg white lysozyme equivalent)/mL), and the protein level (μg/mL). rewrite boxplot description. Statistical information is described in the main text.
(DOCX)
