# Supplementary material for: Immunity for nothing and the eggs for free: Apparent lack of both physiological trade-offs and terminal reproductive investment in female crickets (Gryllus texensis)
Source: PLoS One. 2019 May 15;14(5):e0209957. doi: 10.1371/journal.pone.0209957 (PMC6519836; doi:10.1371/journal.pone.0209957)
Supplement: S1 Methods — (DOCX) [file pone.0209957.s001.docx]

**Supplementary Methods**

**PO and Bradford (total protein) assay**

In this study, we measured total phenoloxidase activity. We also measured the total protein concentration using the same samples. 20 µL of the hemolymph sample (the sample collection procedure is described in the main text) was mixed with 34 µL of reverse-osmosis filtered water (RO water), then spun at 10,000 rpm for 5 minutes at room temperature. 28 µL of the supernatant was mixed with 28 µL of 2 mg/mL chymotrypsin (Sigma, #C7762-100MG) solution, and the mixture was incubated for 20 minutes at room temperature. (The remainder of the hemolymph sample (26µL) was used to measured its protein concentration with a Bradford assay kit (Sigma, #B6916-500ML), following the manufacturer’s instructions.) After the incubation, we spun the sample at 10,000 rpm for 5 minutes at room temperature and the supernatant was used for the assay. We then loaded a 96-well microplate wells with 180 µL of L-3,4-dihydroxyphenylanaline (L-DOPA) solution. The L-DOPA solution (saturated) was prepared by mixing 118 mg of L-DOPA powder (Sigma, #D9628-25G) in 30 mL of RO water. Samples were run as triplicates. 15 µL of samples were run. 15 µL of RO water was run as blanks. A standard curve was determined using tyrosinase (Sigma, #T3824-25KU). Tyrosinase standards for this assay were 450, 45, 22.5, 4.5, 2.25, 0.90, and 0.18 µg powder/mL. Wells were measured at OD490 every 30 seconds for 30 minutes. The enzymatic activity of the lot used in this study was 2687 units/mg solid. A standard curve within the linear range was used for estimating the total phenoloxidase activity of the samples as a tyrosinase equivalent (µg/mL) in each trial.

**GSH assay**

We used a commercial kit (Cayman Chemical, #703302) for GSH measurements. After thawing the deproteinated hemolymph samples, we processed the samples according to the manufacturer’s instructions. We used the protocol for measuring total (i.e. both reduced and oxidized forms of) GSH.

**Gene expression analysis**

For RNA extraction, we used RNeasy Lipid Tissue Mini kit (Qiagen, #74804). After spinning the tissue sample in the RNAlater, we discarded the supernatant, added 1mL of Qiazol (Qiagen, #79306), and homogenized the sample using Tissue Raptor (Qiagen, #9002756). Extracted RNA was then diluted, and 200 ng of the total RNA was then reverse transcribed by using iScript Reverse Transcription Supermix (Bio-Rad, #1708840). RNA was quantified using an Epoch spectrophotometer (BioTek Instruments, Inc., Winooski, VT, USA), and 200 ng of RNA was used for the cDNA synthesis. We diluted the cDNA sample 20 fold by RNase-free water. 4 µL of the diluted cDNA were used for qPCR, mixed with 5 µL of SsoAdvanced™ Universal SYBR® Green Supermix and 0.5 µL each of a forward and a reverse primer (the total reaction volume = 10 µL). Each assay was run on a CFX96 or a CFX384 system (Bio-Rad) depending on the number of samples. All primers were first validated and the amount of cDNA was within linear range. The expression levels were normalized by a geometrical mean of two reference genes (tubulin and cytochrome B). We chose these two reference genes from multiple housekeeping genes using NormFinder (<https://moma.dk/normfinder-software>). qPCRs were done with triplicate samples (3 technical replicates on each biological replicate) with an NTC (no template control) for each biological replicate. No signal was detected in NTC samples. Amplification efficiencies were between 90% and 110%. Amplification specificity was confirmed by melt curve for each reaction.

**Statistical Analysis**

To test the effect of immune challenges on egg count data, we used generalized linear models, assuming negative binomial errors for the response variable. We used a log link function to give a following model:

log($y)= \beta_{0}+\sum_{i} \beta_{i}\cdot X_{i}$

where $y$: egg count, $\beta_{0}$: intercept, $\beta_{i}:$ coefficient for i-th treatment, $X_{i}:$ dummy parameter for i-th treatment (0 or 1). For each statistical tests, we first compared a Akaike’s information criteria (AIC) calculated for a model shown above and that for a model where $\beta_{i}= 0$ for all $i$ (which provides a model without treatment effects). Because the AICs for the model containing treatment effects were greater than the those of null models (see the main text), we did not perform post-hoc analysis.

To test the effect of immune challenges on immune measures, we used generalized linear mixed models, assuming gamma distribution for the response variable. We used Gaussian distribution for GSH on day 12 because the observed data distributed normally. Normality was tested by Shapiro-Wilk test using ‘shapiro.test’ function in R. We used a log link function to give a following model:

log($y)= \beta_{0}+\sum_{i} \beta_{i}\cdot X_{i}$

where $y$: immune measure, $\beta_{0}$: intercept, $\beta_{i}:$ coefficient for i-th treatment, $X_{i}:$ dummy parameter for i-th treatment (0 or 1). For each statistical tests, we first compared a Akaike’s information criteria (AIC) calculated for a model shown above and that for a model where $\beta_{i}= 0$ for all $i$ (which provides a model without treatment effects). Tested models are listed in Table 5 in the main text. If the AIC of the model containing treatment effect is lower than that of its null model, then we performed post-hoc z-test to test whether the coefficient of the fixed effect (treatment effect) is non-zero (i.e. significant difference from reference). We performed eight post-hoc tests in this analysis (see Table 5), and the significance levels were corrected by Benjamini-Hochberg procedure to control the false discovery rate at 0.05. We used ‘glmer’ function in R. Survival analyses (shown in Fig. S2) were performed using ‘survival’ package on R.

To test the effect of age on immune measures, we used generalized linear mixed models, assuming gamma distribution for the response variable. Normality was tested by Shapiro-Wilk test using ‘shapiro.test’ function in R. We used a log link function to give a following model:

log($y)= \beta_{0}+\sum_{i} \beta_{i}\cdot AgeClass_{i}$

where $y$: immune measure, $\beta_{0}$: intercept, $\beta_{i}:$ coefficient for i-th treatment, $X_{i}:$ dummy parameter for i-th treatment (0 or 1). For each statistical tests, we first compared a Akaike’s information criteria (AIC) calculated for a model shown above and that for a model where $\beta_{i}= 0$ for all $i$ (which provides a model without treatment effects). Tested models are listed in Table 6 in the main text. If the AIC of the model containing treatment effects is lower than that of its null model, then we performed a post-hoc z-test to know whether the coefficient is non- zero (i.e. significant difference from reference). We performed four post-hoc tests in this analysis (Table 6), and the significance level was corrected by Benjamini-Hochberg procedure to control the false discovery rate at 0.05. We used ‘glmer’ function in R.
