# Supplementary material for: Immunity for nothing and the eggs for free: Apparent lack of both physiological trade-offs and terminal reproductive investment in female crickets (Gryllus texensis)
Source: PLoS One. 2019 May 15;14(5):e0209957. doi: 10.1371/journal.pone.0209957 (PMC6519836; doi:10.1371/journal.pone.0209957)
Supplement: S1 Table — (DOCX) [file pone.0209957.s002.docx]

### **S1 Table. List of Primers for qPCR.**

| Target | forward (5'-3') | reverse (5'-3') |
| --- | --- | --- |
| *tubulin (reference)* | CAA CAC GTT CTT CAG CGA AA | TTA GTG TAG GTG GGG CGT TC |
| *cytochrome B (reference)* | TGA GGT GGA TTT GCA GTT GA | TTG AAG GTG AAG TAC GGA TGG |
| *proPO 1* | CAT CGC CAG GTA CAA CTT CG | GTC CGT TCC CCT CTA GCA TT |
| *proPO 2* | TCC AGT TCA ACT TCC AGC CA | CAG TAG CTG TGA CCT TGG GA |
| *proPO 3* | ACG GCT CCA TCT TCT CCA AA | CGA AGT TGT ACC TGG CGA TG |
| *vitellogenin 1* | TCT CGC ATG ACT CAC GCT AT | GTG AAC TTC CAC CCT ACG GA |
| *vitellogenin 2* | TTT CGC CCT TGC CTG AAA AT | TTG AAA AGC CAG CAT CCC AG |
| *vitellogenin 3* | CAT GGA ATA GGT TGC CGG TG | CAG CGT TTC TAC CGT TCG AG |
