# Supplementary material for: Immunity for nothing and the eggs for free: Apparent lack of both physiological trade-offs and terminal reproductive investment in female crickets (Gryllus texensis)
Source: PLoS One. 2019 May 15;14(5):e0209957. doi: 10.1371/journal.pone.0209957 (PMC6519836; doi:10.1371/journal.pone.0209957)
Supplement: S2 Table — (DOCX) [file pone.0209957.s003.docx]

**S2 Table. Summary of generalized linear models for the effect of immune challenges on reproductive output**

| Data used for analysis | Model Formulae | Family | AIC |
| --- | --- | --- | --- |
| *NTC  Control(E)  Sham(E)  IC(E) | Eggs laid (d12to36) ~ Treatment | Negative Binomial | 890.17 |
|  | Null Model | Negative Binomial | 885.12 |
| *NTC  Control(L)  Sham(L)  IC(L) | Eggs laid (d22 to 36) ~ Treatment | Negative Binomial | 873.01 |
|  | Null Model | Negative Binomial | 868.34 |
| *NTC  Control(E/L)  Sham(E/L)  IC (E/L) | Eggs in LO** (d36) ~ Treatment | Negative Binomial | 1570.99 |
|  | Null Model | Negative Binomial | 1562.06 |

* Reference (intercept) in each model. No post-hoc test was performed.

**LO: the Lateral Oviducts
