# Supplementary material for: Immunity for nothing and the eggs for free: Apparent lack of both physiological trade-offs and terminal reproductive investment in female crickets (Gryllus texensis)
Source: PLoS One. 2019 May 15;14(5):e0209957. doi: 10.1371/journal.pone.0209957 (PMC6519836; doi:10.1371/journal.pone.0209957)
Supplement: S3 Table — (DOCX) [file pone.0209957.s004.docx]

**S3 Table. Summary of generalized linear mixed models for immune measures**

| Data used for analysis | Model Formulae | Family | AIC | Significant treatment effects (post-hoc analysis) | P value (**) |
| --- | --- | --- | --- | --- | --- |
| *Control(E)  Sham(E)  IC (E) | PO (day 12) ~ Treatment | Gamma | **62.2** | **IC (E): Negative Effect (β= -1.26)**  Sham (E): Not significant | **0.012**  0.30 |
|  | Null Model | Gamma | 62.9 | - | - |
| *Control(L)  Sham(L)  IC (L) | PO (day 22) ~ Treatment | Gamma | 246.1 | Not Tested | - |
|  | Null Model | Gamma | 243.1 | - | - |
| *NTC  Control(E/L)  Sham(E/L)  IC (E/L) | PO (day 36) ~ Treatment | Gamma | 340.0 | Not Tested | - |
|  | Null Model | Gamma | 335.2 | - | - |
| *Control(E)  Sham(E)  IC (E) | GSH (day 12) ~ Treatment | Gaussian | **544.6** | IC (E): Not significant  Sham (E): Trend, Positive (β=0.44) | 0.51  0.035 |
|  | Null Model | Gaussian | 550.5 | - | - |
| *Control(L)  Sham(L)  IC (L) | GSH (day 22) ~ Treatment | Gamma | 593.2 | Not Tested | - |
|  | Null Model | Gamma | 590.0 | - | - |
| *NTC  Control(E/L)  Sham(E/L)  IC (E/L) | GSH (day 36) ~ Treatment | Gamma | 1453.6 | Not Tested | - |
|  | Null Model | Gamma | 1446.6 | - | - |
| *Control(E)  Sham(E)  IC (E) | Lysozyme(day 12) ~ Treatment | Gamma | 115.1 | Not Tested | - |
|  | Null Model | Gamma | 111.7 | - | - |
| *Control(L)  Sham(L)  IC (L) | Lysozyme (day 22) ~ Treatment | Gamma | **161.2** | IC (L): Trend, Positive (β=0.62)  Sham (L): Not significant | 0.025  0.80 |
|  | Null Model | Gamma | 163.5 | - | - |
| *NTC  Control(E/L)  Sham(E/L)  IC (E/L) | Lysozyme (day 36) ~ Treatment | Gamma | 373.4 | Not Tested | - |
|  | Null Model | Gamma | 364.2 | - | - |
| *Control(E)  Sham(E)  IC (E) | Protein (day 12) ~ Treatment | Gamma | **43.8** | IC (E): Trend, Positive (β=0.34)  **Sham (E): Positive Effect (β=0.45)** | 0.055  **0.0067** |
|  | Null Model | Gamma | 46.7 | - | - |
| *Control(L)  Sham(L)  IC (L) | Protein (day 22) ~ Treatment | Gamma | 53.6 | Not Tested | - |
|  | Null Model | Gamma | 52.2 | - | - |
| *NTC  Control(E/L)  Sham(E/L)  IC (E/L) | Protein (day 36) ~ Treatment | Gamma | 194.8 | Not Tested | - |
|  | Null Model | Gamma | 187.3 | - | - |

*reference (intercept) in each model and post-hoc test

** Significance level was adjusted by Benjamini-Hochberg procedure
