# Supplementary material for: Immunity for nothing and the eggs for free: Apparent lack of both physiological trade-offs and terminal reproductive investment in female crickets (Gryllus texensis)
Source: PLoS One. 2019 May 15;14(5):e0209957. doi: 10.1371/journal.pone.0209957 (PMC6519836; doi:10.1371/journal.pone.0209957)
Supplement: S4 Table — (DOCX) [file pone.0209957.s005.docx]

**S4 Table. Summary of generalized linear mixed models for immune measures over age**

| Data used for analysis | Model Formulae | Family | AIC | Significant difference from day 12 (post-hoc analysis) | P value (**) |
| --- | --- | --- | --- | --- | --- |
| *Control (E) – d12  Control (L) – d22  NTC – d36 | PO ~ Age | Gamma | 184.0 | **Day 22: Higher (β=1.14)**  **Day 36: Higher (β=1.05)** | **0.0016**  **0.0034** |
|  | Null Model | Gamma | 189.5 | - | - |
| *Control (E) – d12  Control (L) – d22  NTC – d36 | Lysozyme ~ Age | Gamma | 117.6 | Day 22: Trend Higher (β=1.06)  **Day 36: Higher (β=1.59)** | 0.057  **0.012** |
|  | Null Model | Gamma | 119.6 | - | - |
| *Control (E) – d12  Control (L) – d22  NTC – d36 | GSH ~ Age | Gamma | 513.6 | Not Tested | - |
|  | Null Model | Gamma | 511.3 | - | - |
| *Control (E) – d12  Control (L) – d22  NTC – d36 | Protein ~ Age | Gamma | 509.9 | Not Tested | - |
|  | Null Model | Gamma | 508.5 | - | - |
| *Control (E) – d12  Control (L) – d22  NTC – d36 | PO:GSH ratio ~ Age | Gamma | -177.2 | Not Tested | - |
|  | Null Model | Gamma | -178.7 | - | - |

*reference (intercept) in each model and post-hoc test

** Significance level was adjusted by Benjamini-Hochberg procedures
