# Supplementary material for: Immunity for nothing and the eggs for free: Apparent lack of both physiological trade-offs and terminal reproductive investment in female crickets (Gryllus texensis)
Source: PLoS One. 2019 May 15;14(5):e0209957. doi: 10.1371/journal.pone.0209957 (PMC6519836; doi:10.1371/journal.pone.0209957)
Supplement: S1 Fig — Target genes (transcripts) were identified as described in Materials and Methods. Illustrated here is the sequence query (translated to amino acid sequence) and typical hit results in NCBI’s BLAST search. (A) vitellogenins. (B) proPOs. (DOCX) [file pone.0209957.s006.docx]

### **S1 Figure. Sequence information**

###
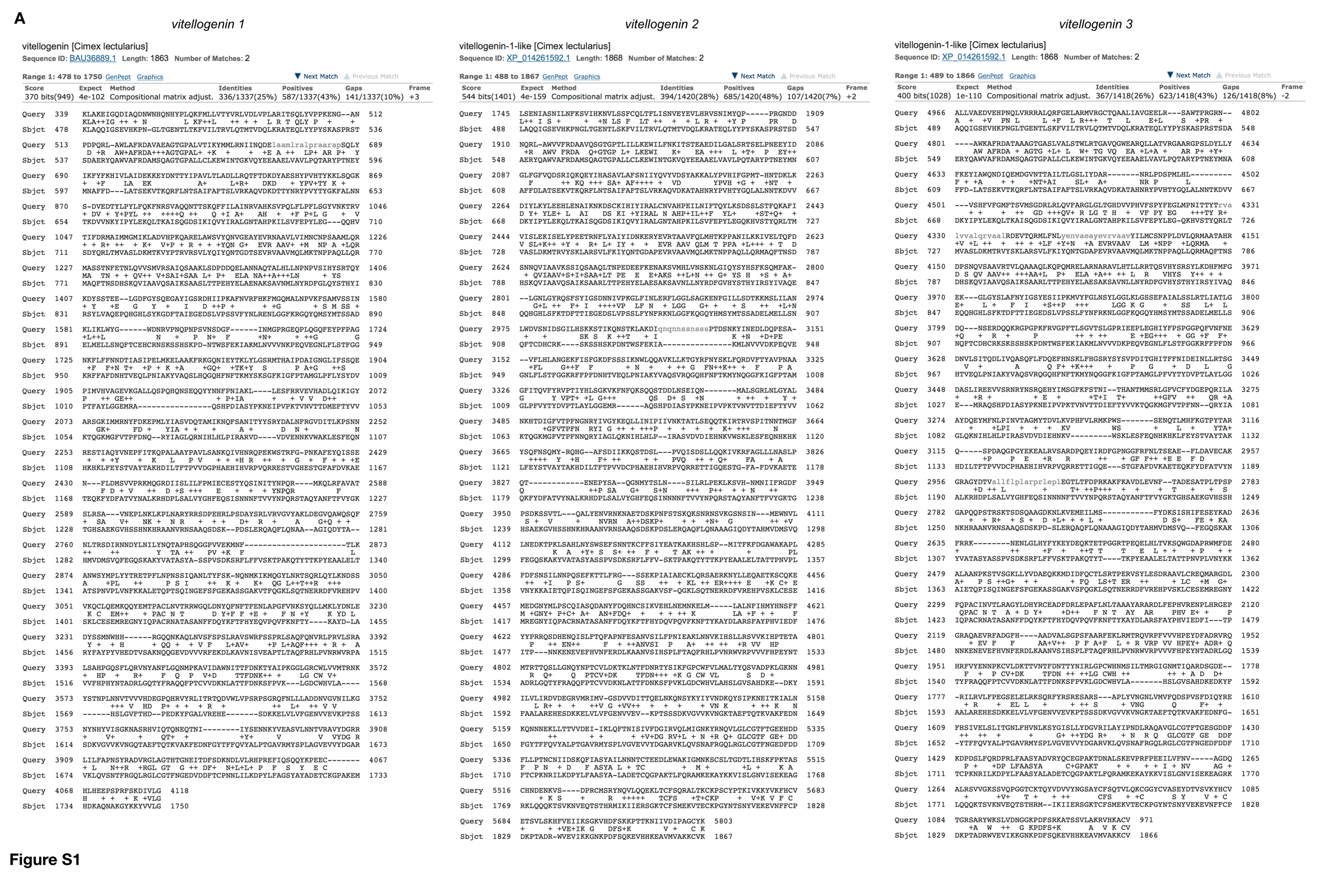


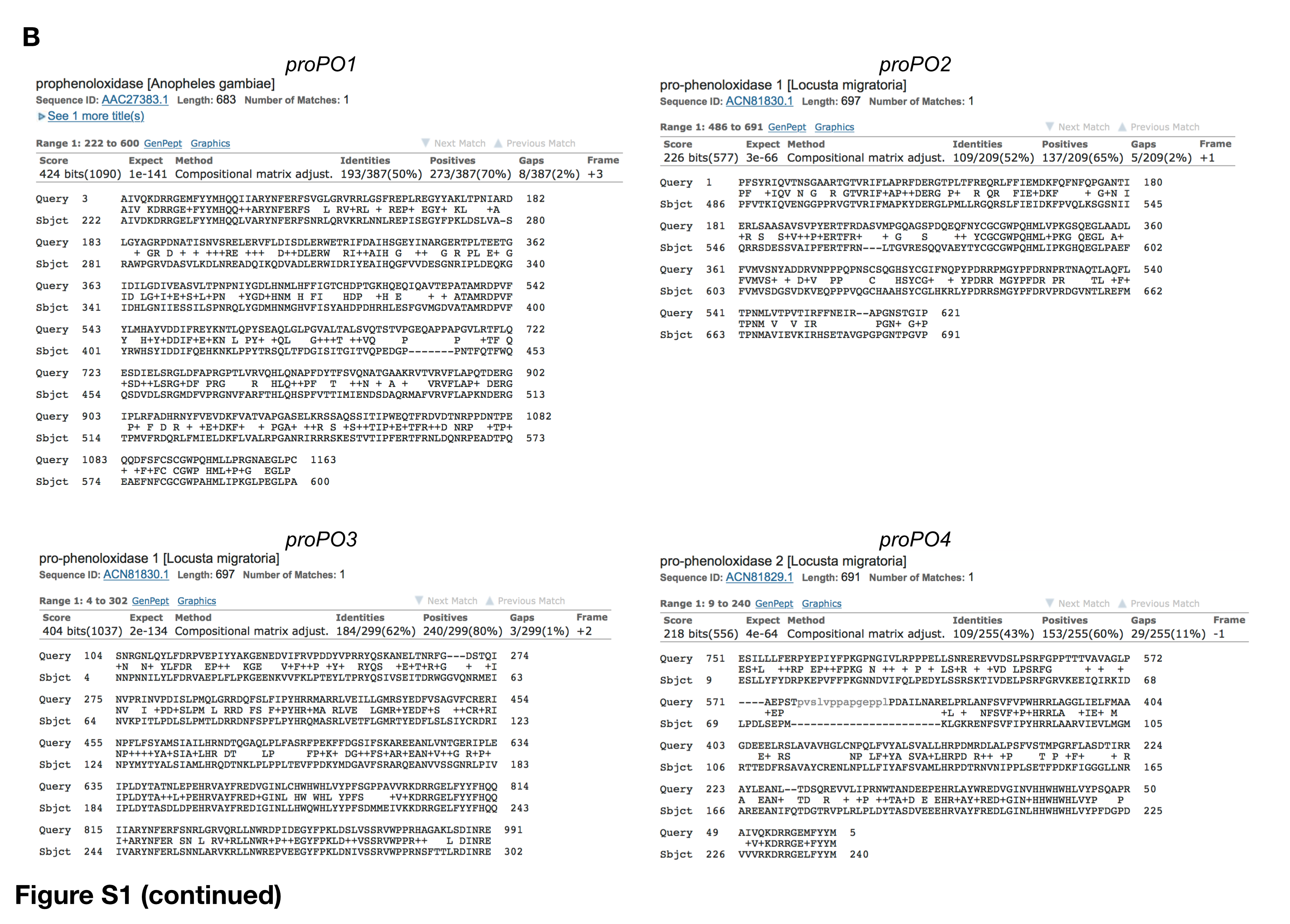


Target genes (transcripts) were identified as described in Materials and Methods. Illustrated here is the sequence query (translated to amino acid sequence) and typical hit results in NCBI’s BLAST search. (A) vitellogenins. (B) proPOs.
