# Supplementary material for: Immunity for nothing and the eggs for free: Apparent lack of both physiological trade-offs and terminal reproductive investment in female crickets (Gryllus texensis)
Source: PLoS One. 2019 May 15;14(5):e0209957. doi: 10.1371/journal.pone.0209957 (PMC6519836; doi:10.1371/journal.pone.0209957)
Supplement: S2 Fig — Survival of crickets. 210 crickets across eight groups (see Materials and Methods) were monitored daily (A). There was no significant difference in survival across the eight groups (B). (DOCX) [file pone.0209957.s007.docx]

### **S2 Figure. Survival**

Survival of crickets. 210 crickets across eight groups (see Materials and Methods) were monitored daily (A). There was no significant difference in survival across the eight groups (B).
