# Supplementary material for: Immunity for nothing and the eggs for free: Apparent lack of both physiological trade-offs and terminal reproductive investment in female crickets (Gryllus texensis)
Source: PLoS One. 2019 May 15;14(5):e0209957. doi: 10.1371/journal.pone.0209957 (PMC6519836; doi:10.1371/journal.pone.0209957)
Supplement: S3 Fig — Physiological validation of the three vitellogenin transcripts (vitellogenin 1, 2, and 3). Normalized expression level in the fat body in females on day 1 (F01), day 12 (F12), day 22 (F22), day 36 (F36) and males on day 12 (M12). Each plot represents an individual cricket (biological replicate). The values in the y-axes represents relative expression levels (arbitrary units), where the expression level for the reference genes is set to be 1.0. (DOCX) [file pone.0209957.s008.docx]

**S3 Figure. Physiological validation of vitellogenin transcripts**


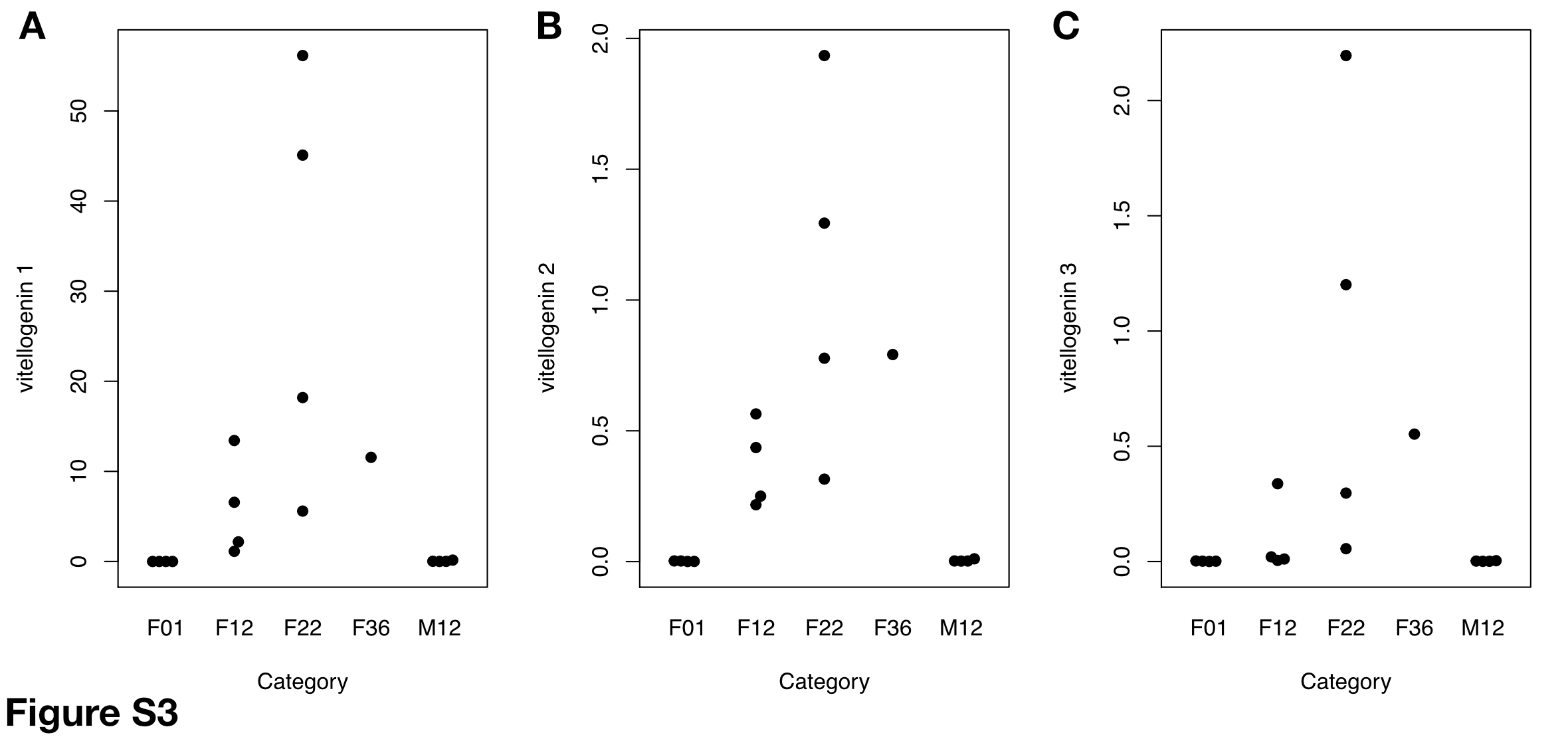


Physiological validation of the three vitellogenin transcripts (vitellogenin 1, 2, and 3). Normalized expression level in the fat body in females on day 1 (F01), day 12 (F12), day 22 (F22), day 36 (F36) and males on day 12 (M12). Each plot represents an individual cricket (biological replicate). The values in the y-axes represents relative expression levels (arbitrary units), where the expression level for the reference genes is set to be 1.0.
