# Supplementary material for: Immunity for nothing and the eggs for free: Apparent lack of both physiological trade-offs and terminal reproductive investment in female crickets (Gryllus texensis)
Source: PLoS One. 2019 May 15;14(5):e0209957. doi: 10.1371/journal.pone.0209957 (PMC6519836; doi:10.1371/journal.pone.0209957)
Supplement: S4 Fig — The number of eggs laid by day 36 (A) and the number of eggs found in the lateral oviducts on day 36 (B) are shown in the chart. Food limited NTC crickets produced fewer eggs compared with ad lib fed NTC crickets The bars represents the 25th and 75th percentile, the central line in bold represents the median and the error bars denote the maximum and minimum values for each group. Statistical information is described in the Results section. (A) Mann-Whitney U test: p = 0.0011. (B) Mann-Whitney U test: p = 7.1x10^-4^. Sample size is n = 20 for each group. (DOCX) [file pone.0209957.s009.docx]

### **S4 Figure. Effect of food limitation on reproductive output**

###

The number of eggs laid by day 36 (A) and the number of eggs found in the lateral oviducts on day 36 (B) are shown in the chart. Food limited NTC crickets produced fewer eggs compared with *ad lib* fed NTC crickets The bars represents the 25^th^ and 75^th^ percentile, the central line in bold represents the median and the error bars denote the maximum and minimum values for each group. Statistical information is described in the Results section. (A) Mann-Whitney U test: p = 0.0011. (B) Mann-Whitney U test: p = 7.1x10^-4^. Sample size is n=20 for each group.
