# Supplementary material for: Immunity for nothing and the eggs for free: Apparent lack of both physiological trade-offs and terminal reproductive investment in female crickets (Gryllus texensis)
Source: PLoS One. 2019 May 15;14(5):e0209957. doi: 10.1371/journal.pone.0209957 (PMC6519836; doi:10.1371/journal.pone.0209957)
Supplement: S5 Fig — We found no significant effect of immune challenge on the reproductive output of female crickets. The parameter estimates in the generalized linear mixed model were: Dispersion parameter for Negative Binomial 0.5187 β for Intercept (β_0) 4.2966 β for IC(L) effect (β_(IC(L))) -0.1302 Using these parameter estimates for gamma distribution, we performed a power analysis by simulation. We iterated 200 random samplings from two gamma distributions (the μ of distribution 1 is exp(4.2966) and that of distribution 2 is exp(4.2966–0.1302)) with different sample sizes (10 to 3000). We calculated the power by dividing the number of positive instance by 200. In other words, if 80 out of 200 iterations found significant difference, then the power is 0.4. The y-axis of the figure represents the calculated power, and the x-axis represents the sample size. As shown in the figure, more than >1700 crickets were required to obtain a power of >0.8. Attached below is the code in R for this computation. (DOCX) [file pone.0209957.s010.docx]

### **S5 Figure. Power analysis of the statistical test for the effect of immune challenge on reproduction**

We found no significant effect of immune challenge on the reproductive output of female crickets. The parameter estimates in the generalized linear mixed model were:

Dispersion parameter for Negative Binomial 0.5187

*β* for Intercept ($\beta_{0})$ 4.2966

*β* for IC(L) effect ($\beta_{IC(L)})$ -0.1302

Using these parameter estimates for gamma distribution, we performed a power analysis by simulation [1]. We iterated 200 random samplings from two gamma distributions (the *µ* of distribution 1 is exp(4.2966) and that of distribution 2 is exp(4.2966 – 0.1302)) with different sample sizes (10 to 3000). We calculated the power by dividing the number of positive instance by 200. In other words, if 80 out of 200 iterations found significant difference, then the power is 0.4. The y-axis of the figure represents the calculated power, and the x-axis represents the sample size. As shown in the figure, more than >1700 crickets were required to obtain a power of >0.8. Attached below is the code in R for this computation.

posi.rate <- c()

sampsize.range <- seq(from = 10, to = 3000, by = 100)

counter <- 1

for (sampsize in sampsize.range){

result <-c()

for (itr in 1:200){

d1<- rnbinom(n=sampsize, size = 0.5187, mu = exp(4.2966))

d2<- rnbinom(n=sampsize, size = 0.5187, mu = exp(4.2966-0.1302))

simd <- cbind (y = c(d1, d2), x =c(rep(1, sampsize), rep(2, sampsize))); simd <- as.data.frame(simd)

res <- glm.nb(y ~ as.factor(x), data =simd, link="log")

pval <- coef(summary(res))[2,4];pval

result <- c(result, pval)

rm (pval, d1, d2, simd, res)

}

pr <-sum(result < 0.05)/length(result)

posi.rate <- c(posi.rate, pr); posi.rate

print(paste ("step", counter, "of", length(sampsize.range), ": posirate =", pr )); counter <- counter +1

rm(result, pr)

}

par(mfrow=c(1,1))

plot (x= sampsize.range, y = posi.rate, type="p",pch=19, ylim=c(0, 1), ylab="Power", xlab="Sample size")

segments(0, 0.8, 3000, 0.8, lty=3)

**Reference**

1. Zhu H, Lakkis H. Sample size calculation for comparing two negative binomial rates. Stat Med. 2014;33: 376–387. doi:10.1002/sim.5947
