# Supplementary material for: Immunity for nothing and the eggs for free: Apparent lack of both physiological trade-offs and terminal reproductive investment in female crickets (Gryllus texensis)
Source: PLoS One. 2019 May 15;14(5):e0209957. doi: 10.1371/journal.pone.0209957 (PMC6519836; doi:10.1371/journal.pone.0209957)
Supplement: S6 Fig — Time course of RV. The reproductive value (RV), a proxy for fitness, was calculated as a product of the number of eggs and the hatch ratio. It provides a rough estimation of the number of offspring for each female. For example, if a female laid 50 eggs and 3 out of the 5 subsampled eggs hatched, then the reproductive value would be 30. Each black line represents an individual cricket. The red lines (with dots) represents median values at each time point. The RVs are log10-transformed. The total sample size for the crickets shown above is 136, because 10 of 146 crickets lacked hatchling data, and, thus, were not possible to obtain RVs. Below is a list of treatments. (DOCX) [file pone.0209957.s011.docx]

### **S6 Figure. Time course of reproductive value (RV) (by treatment group)**

­

Time course of RV. The reproductive value (RV), a proxy for fitness, was calculated as a product of the number of eggs and the hatch ratio. It provides a rough estimation of the number of offspring for each female. For example, if a female laid 50 eggs and 3 out of the 5 subsampled eggs hatched, then the reproductive value would be 30. Each black line represents an individual cricket. The red lines (with dots) represents median values at each time point. The RVs are log10-transformed. The total sample size for the crickets shown above is 136, because 10 of 146 crickets lacked hatchling data, and, thus, were not possible to obtain RVs. Below is a list of treatments.

*Early Treatment Controls (Control (E))*. Crickets were handled on day 11, and hemolymph samples were collected on days 12 and 36.

*Late Treatment Controls (Control (L))*. Crickets were handled on day 21, and hemolymph samples were collected on days 22 and 36.

*Immune Challenge – Early Treatment (IC (E))*. On day 11, crickets were injected with 2 µL of a mixture of heat-killed pathogen cells *(Serratia marcescens*, *Bacillus cereus* and *Beauveria bassiana*.) Bacteria were obtained from Carolina Biological (Microkwik cultures, Burlington, NC, USA) and the *Beauveria bassiana* was BotaniGard 22WP (Laverlam, Butte, MT, USA). The dose of each pathogen was approximately 1/10 of the LD_50_ dose prior to heat inactivation (*S. marcescens*, 2 x10^4^ cells; *B. cereus* 2 x 10^3^ cells; *B. bassiana*, approximately 1 x 10^4^ cells). Hemolymph samples were collected on days 12 and 36. The heat-killed bacteria treatment is known to activate immune responses in this species. We added the fungi, also known to activate immune responses in related insects (e.g. Mormon crickets), to increase the breadth of the challenge.

*Immune Challenge-Late Treatment (IC (L))*. Crickets were injected on days 21 with 2 µL of the same heat-killed pathogen mixture described above. Hemolymph samples were collected on days 22 and 36.

*Sham-Early Treatment (Sham (E))*. On day 11, crickets were poked with a 10 µL Hamilton syringe, but were not injected. Hemolymph samples were collected on days 12 and 36.

*Sham-Late Treatment (Sham (L))*. On day 21, crickets were poked with a 10 µL Hamilton syringe, but not injected . Hemolymph samples were collected on days 22 and 36.

*No Treatment Control (NTC)*. Crickets were not handled or sampled throughout during the 36 day trial. Hemolymph samples were collected on day 36.

*No Treatment Control (ad lib feeding) (NTC (ad lib))*. Crickets were fed ad lib for 36 days. Hemolymph samples were collected on day 36.
