# Supplementary material for: Immunity for nothing and the eggs for free: Apparent lack of both physiological trade-offs and terminal reproductive investment in female crickets (Gryllus texensis)
Source: PLoS One. 2019 May 15;14(5):e0209957. doi: 10.1371/journal.pone.0209957 (PMC6519836; doi:10.1371/journal.pone.0209957)
Supplement: S7 Fig — Most crickets had white (histolysed) flight muscle on day 36 (114 of 131 observations; 5 females lacked muscle observation data). Nevertheless, given that the retention of pink (functional) muscle is associated with dispersion capability of crickets (which is one of the important life-history traits in this species), we performed an additional (i.e. post hoc) analysis to examine the association between the dispersion capability and the reproductive outputs. We excluded the NTC (ad lib) group from this analysis because we did not note wing muscle colour in this group. The crickets that still retained pink (functional) flight muscles on day 36 (n = 17) produced and laid fewer eggs than the crickets with histolysed (white) flight muscle (n = 114). Mann-Whitney U test: W = 303, p-value = 5.2x10^-06^. The bars represents the 25th and 75th percentile, the central line in bold represents the median and the error bars denote the maximum and minimum values for each group. 15 of 146 crickets were excluded from this analysis, because they lacked muscle observation data. (DOCX) [file pone.0209957.s012.docx]

### **S7 Figure. Dispersion capability may be traded off with reproduction**

Most crickets had white (histolysed) flight muscle on day 36 (114 of 131 observations; 5 females lacked muscle observation data). Nevertheless, given that the retention of pink (functional) muscle is associated with dispersion capability of crickets (which is one of the important life-history traits in this species) [1], we performed an additional (i.e. post hoc) analysis to examine the association between the dispersion capability and the reproductive outputs. We excluded the NTC (ad lib) group from this analysis because we did not note wing muscle colour in this group. The crickets that still retained pink (functional) flight muscles on day 36 (n=17) produced and laid fewer eggs than the crickets with histolysed (white) flight muscle (n=114). Mann-Whitney U test: W = 303, p-value = 5.2x10^-06^. The bars represents the 25^th^ and 75^th^ percentile, the central line in bold represents the median and the error bars denote the maximum and minimum values for each group. 15 of 146 crickets were excluded from this analysis, because they lacked muscle observation data.

**Reference**

1. Zera AJ. The Endocrine Regulation of Wing Polymorphism in Insects: State of the Art, Recent Surprises, and Future Directions. Integr Comp Biol. 2003;43: 607–616. doi:10.1093/icb/43.5.607
