# Supplementary material for: Immunity for nothing and the eggs for free: Apparent lack of both physiological trade-offs and terminal reproductive investment in female crickets (Gryllus texensis)
Source: PLoS One. 2019 May 15;14(5):e0209957. doi: 10.1371/journal.pone.0209957 (PMC6519836; doi:10.1371/journal.pone.0209957)
Supplement: S8 Fig — Hemolymph biochemistry parameters are shown in the chart. The columns represent measurements on days 12, 22 or 36. The rows represent PO, GSH, Lysozyme-like activity, and the total protein levels. The treatment effects were examined by generalized linear mixed models (using ‘glmer’ function in 'lme4' package in R), considering cohort (i.e. experimental replicate) as a random factor in the model. The y-axes represent PO level (μg (tyrosinase equivalent)/mL), the GSH level (μM), the lysozyme-like activity (μg (egg white lysozyme equivalent)/mL), and the protein level (μg/mL). rewrite boxplot description. Statistical information is described in the main text. (DOCX) [file pone.0209957.s013.docx]

### **S8 Figure. Humoral responses to immune challenge**

Hemolymph biochemistry parameters are shown in the chart. The columns represent measurements on days 12, 22 or 36. The rows represent PO, GSH, Lysozyme-like activity, and the total protein levels. The treatment effects were examined by generalized linear mixed models (using ‘glmer’ function in 'lme4' package in R), considering cohort (i.e. experimental replicate) as a random factor in the model. The y-axes represent PO level (µg (tyrosinase equivalent)/mL), the GSH level (µM), the lysozyme-like activity (µg (egg white lysozyme equivalent)/mL), and the protein level (µg/mL). rewrite boxplot description. Statistical information is described in the main text.
